# An easy protective measure in ophthalmology against medical supply shortage

**DOI:** 10.1017/ice.2020.136

**Published:** 2020-04-16

**Authors:** Fen Tang, Guangyi Huang, Wei Huang, Fan Xu

**Affiliations:** 1 Department of Ophthalmology, People’s Hospital of Guangxi Zhuang Autonomous Region, Nanning, Guangxi, China; 2 Department of Ophthalmology, Renmin Hospital of Wuhan University, Wuhan, Hubei, China

*To the Editor*—The rapidly spreading pandemic of coronavirus disease 2019 (COVID-19) poses a huge challenge to global public health. Healthcare systems in many countries are facing severe shortages of medical supplies.^1-3^

**Fig. 1.**
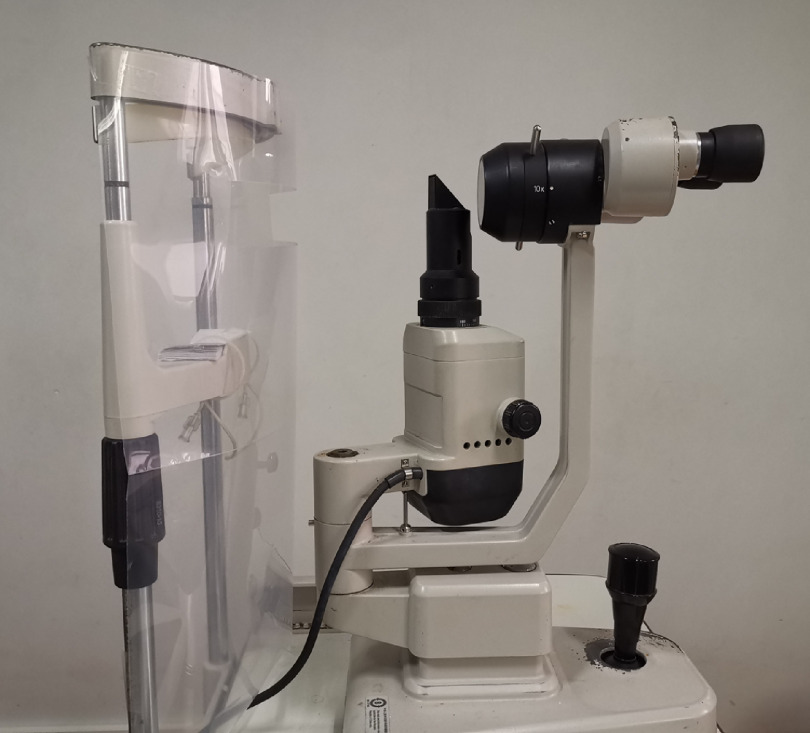
A transparent shield at the slit lamp.

Healthcare workers in ophthalmology departments are susceptible to cross infection because routine ophthalmic examinations, such as like slit-lamp microscopy, are usually performed in a setting of close doctor–patient contact.^4^ Three ophthalmologists in Wuhan Central Hospital were infected with SARS-CoV-2 and died from severe COVID-19.^5,6^ At the ophthalmic center of Guangxi Province, we have continued to provide medical services for outpatients who need timely services during the COVID-19 pandemic. However, in response to the lack of personal protective equipment (PPE) (eg, face shields and gowns) during the epidemic peak in China,^7^ we have adopted an easy measure to perform slit-lamp examinations. After thorough disinfection, we installed a home-made shield at the slit-lamp to separate patients and ophthalmologists. As shown in Figure 1, the protective shield is placed between the chin rest/headrest and the microscope.

The protective shield could prevent contamination of the slit-lamp by respiratory droplets. Although the shortage of medical supplies is currently being alleviated in China, we are encouraging the continuation of this easy protective measure to curb cross transmission in ophthalmology and to protect the ophthalmologists on the frontline of the pandemic.
